# Domestic Summary

**Published:** 1839-06-15

**Authors:** 


### DOMESTIC SUMMARY.

*Medical Institution of Yale College.*—The Annual Circular of this Institution announces a list of seventeen graduates, and of two licentiates in medicine, for the year 1839.

*American Phrenological Journal.*—The June number of this Journal has come to hand, and contains an interesting variety of matter. It is published at $2 per annum, by Mr. A. Waldie, 46 Carpenter street.

